# Supplementary material for: MetaRibo-Seq measures translation in microbiomes
Source: Nat Commun. 2020 Jun 29;11:3268. doi: 10.1038/s41467-020-17081-z (PMC7324362; doi:10.1038/s41467-020-17081-z)
Supplement: Supplementary file 10 — Supplementary Data 7 [file 41467_2020_17081_MOESM10_ESM.zip › File2/Confidence_High_Taxonomy/91192_out.krona.html]

Javascript must be enabled to view this page.

members
magnitude
magnitudeUnassigned
count
unassigned
taxon
rank

91192\_out

68

superkingdom
2
68

68
phylum
976

200643
class
68

68
order
171549

1853231
family
1

1
genus
574697

species
1472416

SRS097889\_contig\_number\_6398
1

family
815
67


SRS013521\_contig\_number\_contig-100\_9013.46349SRS018836\_contig\_number\_10688SRS019910\_contig\_number\_26042SRS021219\_contig\_number\_11857SRS021948\_contig\_number\_contig-100\_286.201535SRS048164\_contig\_number\_contig-100\_341.177810SRS078665\_contig\_number\_contig-100\_327.77160SRS145497\_contig\_number\_17885SRS147271\_contig\_number\_contig-100\_300.233428SRS147557\_contig\_number\_25465SRS149879\_contig\_number\_12281
11
67
genus
816

1

SRS050026\_contig\_number\_10443
species
28111

species
2292931

SRS077127\_contig\_number\_contig-100\_303.116249
1

2292934
species

SRS017103\_contig\_number\_37845SRS024331\_contig\_number\_contig-100\_316.203926
2

1
species
2292936

SRS018623\_contig\_number\_2489

14

SRS017433\_contig\_number\_10922SRS017701\_contig\_number\_9694SRS049712\_contig\_number\_16975SRS062701\_contig\_number\_6676SRS076756\_contig\_number\_9899SRS077231\_contig\_number\_20589SRS1055022\_contig\_number\_18389SRS1055043\_contig\_number\_12049SRS1055099\_contig\_number\_10772SRS142781\_contig\_number\_3320SRS143876\_contig\_number\_30602SRS144362\_contig\_number\_1275SRS893295\_contig\_number\_11206SRS893300\_contig\_number\_5589
1946089
species

1
species
457393

SRS043001\_contig\_number\_1488


SRS143780\_contig\_number\_17579
species
1917883
1

33
820
species

SRS011239\_contig\_number\_3704SRS015190\_contig\_number\_10488SRS015578\_contig\_number\_2466SRS016095\_contig\_number\_15199SRS016132\_contig\_number\_1088SRS017521\_contig\_number\_43039SRS024492\_contig\_number\_523SRS024663\_contig\_number\_1195SRS042284\_contig\_number\_1087SRS043411\_contig\_number\_11342SRS049402\_contig\_number\_8974SRS049995\_contig\_number\_25739SRS050998\_contig\_number\_4937SRS055017\_contig\_number\_2600SRS063190\_contig\_number\_10622SRS075716\_contig\_number\_1280SRS077502\_contig\_number\_5831SRS078176\_contig\_number\_17256SRS104084\_contig\_number\_4133SRS1041118\_contig\_number\_4204SRS1041142\_contig\_number\_318SRS1041145\_contig\_number\_36316SRS1055038\_contig\_number\_49SRS1055067\_contig\_number\_8751SRS140492\_contig\_number\_6574SRS143466\_contig\_number\_25981SRS143598\_contig\_number\_11540SRS893172\_contig\_number\_10634SRS893187\_contig\_number\_contig-100\_175.103950SRS893253\_contig\_number\_81SRS893256\_contig\_number\_731SRS893292\_contig\_number\_2232SRS971275\_contig\_number\_22487

2
species
28116

SRS019693\_contig\_number\_1851SRS053649\_contig\_number\_3262
